# Motivation in medical students: higher intrinsic motivation among graduate-entry students across academic stages

**DOI:** 10.3389/fmed.2025.1625352

**Published:** 2025-06-25

**Authors:** Rita Matos Sousa, Maurílio Santos, Marco Marangoni, Vitor Hugo Pereira

**Affiliations:** ^1^School of Medicine, University of Minho, Braga, Portugal; ^2^Clinical Academic Center-Braga (2CA), Braga, Portugal; ^3^Centro Universitário Max-Planck (UniMAX), Indaiatuba, SP, Brazil; ^4^UNESP—São Paulo State University—Medical School, Botucatu, SP, Brazil

**Keywords:** medical education, academic motivation, self-determination theory, graduate entry, intrinsic motivation

## Abstract

Academic success and professional development are determined by several factors, being motivation an important one. In this study we explored its different dimensions of motivation among students enrolled in the undergraduate medical degree at the School of Medicine of the University of Minho, comparing students from the traditional national entry and graduate entry (PA) pathways across all curricular years. A total of 354 students (response rate: 39.4%) participated in this cross-sectional study during the 2023/2024 and 2024/2025 academic years. Motivation was assessed using the Minho Medical Academic Motivation Scale—Minho-MEDAMS, a validated scale adapted within the Self-Determination Theory framework. Motivation types examined included intrinsic motivation (IM), identified regulation (EMID), introjected regulation (EMIR), external regulation (EMER), and amotivation (AMOT), alongside the Self-Determination Index (SDI). Statistical analyses were conducted to compare motivation across academic years, entry pathways, gender, and age groups. EMID and IM were the most prevalent motivation types, while AMOT was the least reported. No significant differences were found in motivation levels across academic years. PA students showed significantly higher levels of IM, EMID, and SDI compared to traditional pathway students. Motivation levels did not vary significantly with age, and gender differences were minimal, with the exception of higher EMER scores among male students. In conclusion, these findings suggest that graduate-entry students are more self-determined in their motivation profiles, independent of age, and that motivation remains relatively stable throughout the medical curriculum.

## Introduction

Student engagement and academic performance are critically determined by motivation, particularly in demanding programs such as medicine. Several studies (1, 2) have demonstrated that the way students face learning, develop their learning strategies is dependent on their motivation levels; the same holds true for other dimensions such as their resilience, well-being, and future professional behavior. Given its relevance in all these dimensions, the understanding of motivational dynamics amongst medical students is critical for several strategies in medical education that span from the curriculum design, to the profile of entry pathways.

At the School of Medicine of the University of Minho (EM-UMinho), students enter the medical program through two distinct pathways: the traditional national access route, targeting students directly from secondary school, and the graduate entry program, aimed at students who already hold a prior university degree. These cohorts often differ in terms of age, academic and life experience, and career expectations—factors that are known to influence motivational orientations (1, 3).

One of the most consolidated trends in the field of medical education is the move to student-centered pedagogical approaches that promote active learning through clinical cases integrated across biomedical and clinical sciences (4–6). The EM-UMinho’s curriculum, for example, follows this trend and proposes to medical students a curriculum grounded in case-based learning that promotes active student engagement in the acquisition of multiple competencies. In light of the Self-Determination Theory (SDT), such approaches aim to stimulate intrinsic motivation (IM) by emphasizing relevance, autonomy, and contextual learning (5, 7, 8). Notwithstanding these facts, it is still not completely known how the dynamics of motivation shift in response to changing academic demands, exposure to real-world medical practice, and evolving professional identity, namely as students’ progress from more pre-clinical foundational learning to clinical immersion during the general internship (9, 10).

To investigate these dynamics in motivation, we employed a validated instrument specifically adapted for the Portuguese medical education context: Minho Medical Academic Motivation Scale—Minho-MEDAMS, which was designed to assess medical students’ motivation within the multidimensional motivational framework based on SDT (9), and has demonstrated strong psychometric properties (11). This scale allows the exploration of various motivational constructs, including IM, identified regulation (EMID), introjected regulation (EMIR) and external regulation (EMER), and amotivation (AMOT), providing a comprehensive picture of students’ motivational profiles across different stages of their education.

This study aims to deepen the understanding of medical student motivation by exploring differences in motivational profiles between students from distinct entry pathways at the EM-UMinho. While prior studies have investigated motivation in medical education, few have examined how it varies across entry routes or academic stages within a single curriculum. Using the Minho-MEDAMS, this study provides a cross-sectional analysis that (1) identifies motivational patterns associated with graduate-entry students and (2) examines whether motivation appears stable or variable across different years of the program. Although not longitudinal, this approach offers insight into how student motivation may relate to academic background and progression within a consistent institutional setting.

## Materials and methods

### Study design

A correlational, cross-sectional study was conducted to explore associations between demographic variables (age, gender, and school year) and medical students’ motivation levels. Data collection spanned 15 months, from November 2023 to March 2025, and took place at the EM-UMinho.

The experimental protocol was approved by the Ethics Committee of the University of Minho (CEICVS-121/2023). Adherence to the Helsinki Declaration and the Convention on Human Rights of the Council of Europe was strictly observed. Written informed consent was obtained from each participant before data collection.

### Context and participants

All medical students enrolled at EM-UMinho (approximately 900 students) across six academic years were eligible to participate. Recruitment was conducted via institutional email and in-person seminars, where a dedicated website was shared containing demographic questions and the Minho-MEDAMS (11). Access was provided through direct links and QR codes.

In Portugal, medical education follows a 6-year integrated master’s degree model, beginning immediately after secondary school. At the EM-UMinho, students may enter through the traditional national access pathway, or through the graduate-entry (PA) program, which is reserved for applicants who hold a prior university degree in a health-related field. The traditional entry route is for secondary school graduates admitted through the national higher education selection system based on academic performance, with approximately 120 students per year. The PA curriculum condenses the first 3 years into one and provides increased clinical exposure from the outset, promoting early integration into medical settings. Its students are selected through a competitive process involving written exams and interviews, coordinated by EM-UMinho faculty. This pathway is limited to approximately 18 students per year—the PA students.

PA students complete foundational medical sciences in a single year, whereas traditional students cover the same content over 2 years and also have one additional year of elective coursework. From the clinical phase onward, all students follow a unified curriculum, regardless of their entry route.

For the purposes of this study, students were grouped according to academic year (1st through 6th year), and PA students were treated as a distinct group under the label “PA.”

### Sample size

To ensure sufficient power for comparing motivation levels between the PA program and the traditional entry route groups, *a priori* sample size estimation was performed using G*Power 3.1. Although the study employed non-parametric tests due to the distribution of the data, the estimation was based on the parametric equivalent—an independent samples *t*-test—which provides a conservative reference. Assuming a medium effect size (Cohen’s *d* = 0.5), a significance level of α = 0.05, and statistical power of 0.80, with an allocation ratio of 1:6 (PA program:traditional students), the minimum required sample size was calculated to be 189 participants (27 in the PA program group and 162 in the traditional group). Our final sample included 30 PA students and 324 traditional-entry students, exceeding the minimum required and thereby ensuring adequate power to detect meaningful differences between groups.

### Measures

The Minho-MEDAMS was used to assess students’ motivational profiles (11). This scale assesses motivation levels among medical students. It is an adaptation of the Academic Motivation Scale, grounded in the SDT and the Self-Efficacy Theory. It comprises 18 items across five motivation dimensions: IM, EMID, EMIR, EMER, and AMOT. Demonstrating strong psychometric properties (Cronbach’s α = 0.831) and construct validity confirmed through factor analysis, Minho-MEDAMS offers a reliable and context-sensitive tool for evaluating medical students’ motivational profiles (11). To assess the internal consistency of the Minho-MEDAMS in the current sample, the Cronbach’s alpha was calculated with a value of 0.804, indicating good internal consistency across all items. These results support the internal reliability of the Minho-MEDAMS when applied to this population.

Additionally, we collected information regarding the age, gender and school year of the students.

With this information, we created several variables:

- Direct variables: age, gender, academic year, and individual item scores of the Minho-MEDAMS.
- Computed variables: composite scores for IM – average score for items 2, 6, 9, 11, 16, and 23, EMID—average score for items 3, 17, and 24, EMIR—average score for items 7, 21, and 28, EMER – average score for items 8, 14, and 15, AMOT—average score for items 5, 12, and 26, and a Self-Determination Index (SDI) – composite score calculated with the following formula: (2 × IM) + (EMID) – [(EMIR + EMER)/2 + (2 × AMOT)].

### Statistical analyses

Descriptive statistics were used to summarize the sample characteristics. Categorical variables were presented as frequencies and percentages, and continuous variables were reported using medians and interquartile ranges (IQR), given the non-normal distribution of the data.

Before proceeding with the analysis, all responses were reviewed to ensure data integrity. In cases of repeated submissions from the same student, only the first complete response was included in order to preserve the independence of observations.

Non-parametric tests were used for all inferential analyses due to the distribution of the data. Comparisons of motivation scores were made across several groups, including academic years, entry pathways (traditional vs. PA), gender, and between the two academic years during which data were collected. The Mann–Whitney U test and the Kruskal–Wallis H test were used for group comparisons, and Spearman’s rank-order correlation was applied to explore associations between age and motivation.

To account for multiple comparisons and reduce the risk of type I error, Bonferroni correction was applied where appropriate. All statistical analyses were conducted using IBM SPSS Statistics, Version 30.0, with a significance level set at *p* < 0.05.

## Results

A total of 354 medical students from the EM-UMinho participated in the study, corresponding to an approximate response rate of 39.4%. Of these, 150 students (42.4%) were from the 1st year, 36 (10.2%) in the 2nd year, 27 (7.6%) in the 3rd year, 54 (15.3%) in the 4th year, 42 (11.9%) in the 5th year and 45 (12.7%) in the 6th year. Of the overall sample of students, 30 (8.5%) were enrolled in the PA program.

The majority of participants were female (*n* = 278; 78.5%), and the median age was 20 years (IQR = 4.0). A total of 264 responses were collected during the 2023/2024 academic year and 90 during 2024/2025. These informations are further detailed in Table 1.

**TABLE 1:** Demographic characteristics of the sample.

Variable	N (%) or Median (IQR)
Participants	354 (100%)
Female	278 (78.5%)
Male	76 (21.5%)
Age (years)	20 (IQR = 4.0)
**Academic year**	
1st	150 (42.4%)
2nd	36 (10.2%)
3rd	27 (7.6%)
4th	54 (15.3%)
5th	42 (11.9%)
6th	45 (12.7%)
PA-Year	30 (8.5%)
**Entry pathway**	
Traditional	324 (91.5%)
PA	30 (8.5%)

**FIGURE 1:**
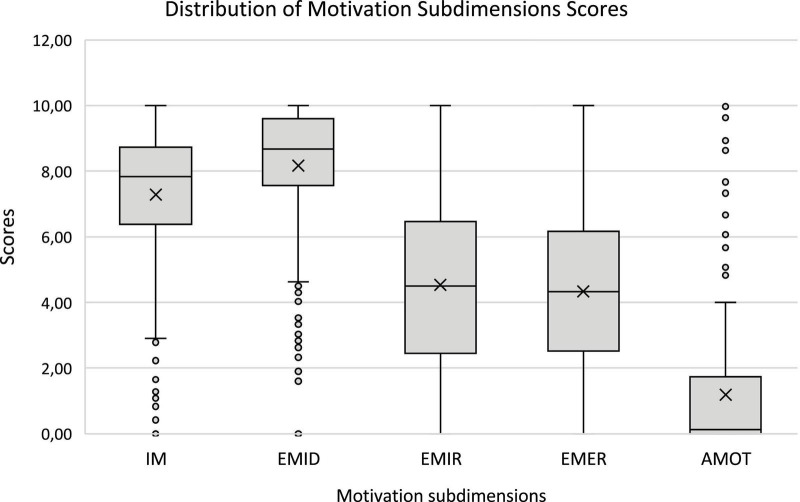
Tukey-style boxplot illustrating the distribution of motivation scores across the five Minho-MEDAMS subdimensions: Intrinsic Motivation (IM), Identified Regulation (EMID), Introjected Regulation (EMIR), External Regulation (EMER), and Amotivation (AMOT).

Overall, when comparing the different types of motivation, median scores were highest for EMID (8.67; IQR = 2.20) and IM (7.78; IQR = 2.40), while AMOT showed the lowest median (0.10; IQR = 1.68). The widest interquartile range was observed for EMIR (IQR = 4.14), indicating greater variability in this dimension, followed by EMER (IQR = 3.70) (Figure 1).

To determine whether motivation levels varied across academic years, a comparative analysis was conducted. No significant differences were found between the two academic years (2023/2024 and 2024/2025) in any of the motivation subdimensions (Table 2).

**TABLE 2:** Comparison of motivation levels between the 2023/2024 and 2024/2025 academic years.

Motivation type	2023/2024 (Median, IQR)	2024/2025 (Median, IQR)	U statistic	p-value
IM	7.8333, 2.31	7.7750, 2.50	12054.0	0.836
EMID	8.6833, 2.01	8.5333, 2.23	11586.5	0.726
EMIR	4.7000, 3.67	4.1000, 4.81	11313.0	0.499
EMER	4.3333, 3.71	4.2167, 3.73	12188.0	0.713
AMOT	0.0500, 1.83	0.3333, 1.67	12212.0	0.674
SDI	17.2500, 9.13	16.9583, 8.22	11905.0	0.976

Similarly, when analyzing motivation levels across the six curricular years, no statistically significant differences were observed (Table 3).

**TABLE 3:** Motivation levels across academic years (1st–6th year).

Motivation type	1st year (Median, IQR)	2nd year (Median, IQR)	3rd year (Median, IQR)	4th year (Median, IQR)	5th year (Median, IQR)	6th year (Median, IQR)	Test statistic	*P*-value
IM	7.6667, 2.52	8.2000, 1.62	8.0333, 2.17	7.9000, 2.15	7.7583, 3.61	7.4000, 3.33	5.647	0.342
EMID	8.5667, 2.27	8.6833, 1.33	8.4667, 1.90	8.8167, 2.03	8.7667, 2.50	8.6333, 2.45	1.082	0.956
EMIR	4.4667, 4.62	4.6333, 4.58	5.3667, 3.17	4.7500, 3.40	4.6500, 4.07	4.0000, 3.70	2.231	0.816
EMER	4.3500, 3.29	4.4500, 4.13	4.7333, 3.57	4.2333, 4.16	3.4000, 4.72	3.8333, 3.12	5.863	0.320
AMOT	0.0000, 1.34	0.0000, 0.92	0.3333, 2.33	0.4000, 1.86	0.3500, 2.80	0.5000, 2.00	5.765	0.330
SDI	17.3250, 9.54	18.9917, 7.20	15.8000, 9.98	16.9750, 8.30	16.1333, 10.08	17.6333, 9.31	3.983	0.552

To explore other possible influencing factors, a comparison was made between students from the traditional entry pathway and those from the PA program. This analysis revealed that PA students presented significantly higher levels of IM, EMID and SDI when compared to students from the traditional pathway (Table 4).

**TABLE 4:** Comparison of motivation levels between traditional and PA program students.

Motivation type	Traditional (Median, IQR)	PA (Median, IQR)	U statistic	*P*-value
**IM**	7.7167, 2.56	8.3167, 1.61	6204.0	0.012
**EMID**	8.6167, 1.99	9.3333, 1.6	6431.0	0.003
EMIR	4.4333, 3.97	5.3333, 3.52	5423.0	0.294
EMER	4.3333, 3.69	4.3000, 3.09	4700.5	0.766
AMOT	0.1167, 1.82	0.2667, 1.08	4696.5	0.746
**SDI**	16.8583, 9.24	20.3583, 6.71	6178.5	0.014

To assess whether this pattern was present from the beginning of the medical course, a further comparison was conducted between 1st-year traditional students and PA students in their first year. Results confirmed the trend, with PA students scoring significantly higher in IM (*p* = 0.001), EMID (*p* = 0.002), and SDI (*p* = 0.020) (Table 5).

**TABLE 5:** Comparison of motivation levels between 1st-year traditional and 1st-year PA program students.

Motivation type	1st year (Median, IQR)	1st year PA (Median, IQR)	U statistic	*P*-value
**IM**	7.5500, 2.77	9.0333, 1.08	503.500	0.001
**EMID**	8.3667, 2.30	9.8667, 1.00	509.000	0.002
EMIR	4.3000, 4.73	5.3333, 4.00	718.000	0.065
EMER	4.3333, 3.50	4.3667, 3.00	958.000	0.733
AMOT	0.0000, 1.40	0.3333, 1.00	1020.500	0.957
**SDI**	16.5000, 10.22	20.6833, 6.18	642.500	0.020

Considering that PA students have previous academic background, we hypothesized that those students would be older than those in the traditional pathway, which was confirmed by a Mann–Whitney *U*-test (*U* = 9414.500, *p* < 0.001), with PA students showing a higher median age (Median = 26.0, IQR = 11) compared to traditional students (Median = 20.0, IQR = 4.0). For this reason, we investigated whether age could act as a confounding factor. On a first analysis, the correlation between age and motivation levels was assessed using Spearman’s correlation coefficient (Table 6). No statistically significant associations were found.

**TABLE 6:** Spearman’s correlations between age and motivation subdimensions.

Correlations	Number	Spearman’s rho	*P*-value
IM-age	354	0.061	0.253
EMID-age	354	0.092	0.083
EMIR-age	354	0.020	0.715
EMER-age	354	−0.070	0.189
AMOT-age	354	0.050	0.348
SDI-age	354	0.041	0.440

Even though the correlations were not significant between age and the different motivation types, we performed a Linear Regression model to better understand if age could in fact be a confounding founder for the PA program students. On this analysis, we confirmed that age is not a significant contributor for motivation levels (Table 7).

**TABLE 7:** Linear regression models predicting motivation from entry pathway and age.

Predictor	B	SE	β	*P*-value
**IM**				
Constant	7.688	0.781		<0.001
PA	1.235	0.517	0.168	0.018
Age	−0.024	0.038	−0.044	0.531
Model fit: R^2^ = 0.020, Adjusted R^2^ = 0.015, *p* = 0.027				
**EMID**				
Constant	8.179	0.724		<0.001
PA	1.014	0.480	0.148	0.035
Age	−0.005	0.035	−0.010	0.891
Model fit: R^2^ = 0.020, Adjusted R^2^ = 0.015, *p* = 0.028				
**SDI**				
Constant	16.722	3.025		<0.001
PA	4.167	2.005	0.146	0.038
Age	−0.055	0.148	−0.026	0.712
Model fit: R^2^ = 0.017, Adjusted R^2^ = 0.011, *p* = 0.049				

Finally, a gender-based analysis was performed. No significant differences were observed between male and female students across most motivational subdimensions, with the exception of EMER, where male students scored significantly higher (Table 8). This suggests a greater influence of external motivational factors, such as social recognition or tangible rewards, among male students.

**TABLE 8:** Comparison of motivation levels by gender.

Motivation type	Female (median, IQR)	Male (Median, IQR)	U statistic	*P*-value
IM	7.8333, 2.33	7.7667, 2.99	10926.000	0.647
EMID	8.7000, 2.14	8.4000, 1.74	11458.000	0.257
EMIR	4.6500, 3.91	4.3167, 4.13	11489.500	0.242
**EMER**	4.000, 3.67	5.3000, 3.56	7888.500	<0.001
AMOT	0.0667, 1.68	0.3500, 1.98	9839.000	0.330
SDI	17.6000, 8.81	15.7333, 9.65	11552.000	0.211

## Discussion

The current study used the Minho-MEDAMS (11), to provide a comprehensive exploration of the dynamics of the motivational dimensions of medical students at the EM-UMinho. The findings herein reported contribute to the growing body of evidence suggesting that medical students’ motivation profiles are nuanced, and shaped, not only by individual factors but also by curricular structures and entry pathways that are offered by institutions; importantly, they inform on the strategic decision-making of pedagogic policies of medical schools.

Across the sample obtained in a single institution, IM and EMID emerged as the most prominent forms of motivation. This finding is in accordance with previous studies showing that autonomous motivation, which includes IM and EMID, is a critical driver for the selection of medicine for future professional career and that choice is primarily guided by internal values and genuine interest, rather than external pressure or obligation (9). Moreover, it also supports the notion that autonomous motivation is a contributor to academic success and well-being during medical training (1, 2). In contrast, the relatively lower levels of EMIR and EMER, along with minimal AMOT, suggest that medical students experience low degrees of controlled motivation and disengagement. Previous studies have also found similar motivational profiles and demonstrated that such profiles are associated with better learning outcomes and student satisfaction in medical education contexts (12). Importantly, other studies have shown also a slightly distinct pattern, and have shown that, in particular contexts, extrinsic motivation is the main determinant of academic success (13).

One of the major findings of the current study, is the demonstration in a similar educational setting, that students from the graduate-entry PA program demonstrated significantly higher scores in IM, EMID, and the SDI compared to traditional-entry students. Importantly, this trend held even when comparing first-year students across both entry pathways, suggesting these differences cannot be solely attributed to time spent in medical school, but rather they represent a trait of the selection process. It is known that there are several factors that enhance internalized and self-determined motivation, such as greater self-awareness, clearer career commitment, and more informed decision-making (2, 3), and such characteristics are more recognized in graduate-entry students.

Our findings align with previous research indicating that graduate-entry students often demonstrate higher levels of self-determined motivation compared to traditional-entry students. This has been observed across various educational contexts, including studies in Ireland (14), Australia (15), the United Kingdom (16, 17), and the United States (18). These studies collectively suggest that prior academic and life experience may contribute to increased intrinsic and identified motivation. Similarly, Feeley and Biggerstaff (19) discuss how learning styles and approaches differ between graduate- and school-leaver entry students, which may also influence motivational patterns. These international comparisons help contextualize our findings and suggest that, despite cultural and structural differences in medical education systems, certain motivational trends may be broadly consistent. For instance, Dodds et al. (15) and Shehmar et al. (17) noted that graduate-entry students often outperform their undergraduate-entry peers in academic performance and engagement, potentially linked to higher intrinsic motivation. Additionally, Sulong et al. (14) highlighted the role of financial and social pressures in shaping extrinsic motivation in Malaysian students, while DeWitt et al. (18) examined burnout patterns associated with entry routes, reinforcing the relevance of motivational profiles in student wellbeing. These studies underscore the importance of curricular and institutional factors, which merit further investigation.

Finally, while this study focuses on a Portuguese context, its findings can be situated within a broader global discussion on medical student motivation. Similar studies in Asia (14), North America (18), and Africa (10) also report higher levels of intrinsic motivation among graduate-entry students or those with prior academic maturity. Cultural expectations, educational models, and healthcare systems appear to influence the types of motivation students exhibit. For example, studies in Malaysia and Ireland have shown that financial burden, societal pressure, and job security play a stronger role in shaping extrinsic motivation (14), while studies in the U.S. highlight the role of autonomy and early clinical exposure in supporting intrinsic motivation (18). These findings align with our observations and suggest that although specific influences vary, self-determined motivation remains a core factor in medical education outcomes globally.

Another important finding of the current study relates to the absence of significant motivational differences across the six curricular years or between the academic years of 2023/2024 and 2024/2025. This suggests a relative stability in motivation levels over time, which is supported by autonomy-promoting educational environments, as explained by the SDT, which posits that motivation, particularly autonomous forms such as IM and EMID, can reflect enduring personal orientations rather than fleeting states (9, 20). It is important to note, however, that there conflicting results on this topic: while there are reports of declining motivation throughout the medical course, often due to increasing stress, clinical fatigue, and a disconnect between coursework and real-world practice (21), there is also evidence that as students progress and gain academic maturity, their professional goals become more clearly defined, facilitating the internalization of values and stabilization of motivation through reflection and identity formation (3). Thus, the current observation of stability in motivation levels throughout the medical course is likely a reflection of both individual dispositional traits and a learning environment that consistently nurtures psychological needs and educational relevance.

Interestingly, no significant gender-based differences were identified in most motivational dimensions, except in EMER, where male students scored higher, suggesting a slightly stronger influence of extrinsic motivators such as prestige or financial incentives. Research in educational psychology has consistently shown gender differences in motivational drivers, with male students often demonstrating a greater sensitivity to extrinsic motivators such as prestige, status, and financial incentives. These patterns are rooted in both sociocultural and psychological frameworks. For instance, Eccles’ expectancy-value theory posits that individuals are influenced by the value they place on different outcomes, and men tend to place higher value on external achievements and tangible rewards due to societal expectations and gender role socialization (22). In medical education, studies have found that male students report stronger extrinsic motivation, particularly EMER through rewards, recognition, or career advancement opportunities (1, 3). Furthermore, research suggests that male students may perceive higher utility in medicine to achieve financial success or social standing, whereas female students are more likely to prioritize intrinsic and altruistic values, such as helping others or personal interest in the subject (23, 24). These tendencies may reflect broader societal structures that associate masculinity with competitiveness and success, reinforcing the pursuit of extrinsically valued goals. Consequently, the higher scores observed in external motivation among male medical students are consistent with existing literature and underscore the role of gendered motivational patterns in educational contexts.

In the current study age was not found to significantly correlate with motivation. This finding may be viewed as surprising, as literature suggests that chronological age can influence motivation levels in university students, although the relationship is nuanced and context dependent. Older students are often more intrinsically motivated than their younger counterparts, likely due to increased maturity, clearer academic goals, and more deliberate educational engagement (25). Studies grounded in SDT also indicate that age is positively associated with autonomous forms of motivation, such as IM and EMID, which are linked to better academic outcomes and persistence (1, 26). While extrinsic motivation may decrease with age in terms of social rewards or peer approval, it can remain stable or increase when tied to career advancement or financial incentives (27). However, these trends are often influenced by associated life circumstances such as employment status, family responsibilities, and previous academic experiences, making it difficult to isolate the effect of age *per se* (28). Therefore, while chronological age appears to shape motivation profiles, it does so in interaction with broader psychosocial and contextual factors. Importantly, it is relevant to highlight that the motivational differences observed in PA students are likely not attributable to age alone. This reinforces the idea that the nature of the entry pathway and prior academic background may be more important than chronological age in influencing motivational orientation (3, 14).

As with any study, this research has several limitations that should be acknowledged. First, the cross-sectional design in a single institution limits the ability to infer causality or assess changes in motivation over time, preventing conclusions about developmental trends or the long-term effects of curricular experiences. Second, the reliance on self-reported data introduces the possibility of social desirability bias and subjective interpretation of the scale items, which may affect the accuracy of the responses. Students may also have inaccurately perceived or reported their motivation due to personal bias or misunderstanding of the questions. Third, although the sample size of 354 students is statistically robust, the response rate of 39.4% is relatively low and may introduce response bias. It is possible that students who were more motivated or engaged were more likely to participate, which could limit the generalizability of the findings to the entire student population. However, the demographic and academic distribution of our participants (by entry route and academic year) closely mirrors the proportions within the medical school’s enrolled population, which supports the relevance and representativeness of our sample. Nonetheless, caution is warranted when generalizing the findings beyond this institutional context. Educational systems, entry routes, and curricular models vary significantly across countries. As this study was conducted within a Portuguese medical school, results may not fully reflect motivational patterns in contexts with different program structures, admission criteria, or cultural norms. Future research should explore cross-national comparisons to validate and extend these findings. Additionally, while we examined demographic factors such as age, gender, and academic year, the study did not include other potentially influential variables such as socioeconomic status, mental health status, or prior academic performance. These factors could have a meaningful impact on students’ motivation and should be considered in future research. The timing of data collection may also have influenced the results, as surveys administered during particularly stressful academic periods (e.g., examination seasons) might reflect transient motivational states rather than stable traits. Moreover, the relatively small number of students from the PA (graduate entry) program, although proportional in terms of response rate, limits the strength of subgroup comparisons. Finally, the study’s findings may not be generalizable to medical schools with different curricular models, admission pathways, or cultural contexts. Despite these limitations, the study provides valuable insights into motivational dynamics in medical education and highlights the importance of considering entry pathways and institutional structures when designing learner-centered educational strategies.

While this study does not directly assess curricular components or educational interventions, the findings open space for reflection on how motivation might be better supported within diverse medical student populations. For instance, traditional-entry students, who may be younger and less experienced, might benefit from strategies that promote early autonomy, relevance in learning, and personal engagement—elements known to support the development of intrinsic motivation. In contrast, graduate-entry students, who often begin with higher levels of self-determination, may benefit from flexible and personalized learning environments that sustain their internal drive. Although further research is needed to explore the effectiveness of such approaches, these reflections may help inform discussions around curriculum development and student support in ways that are responsive to different motivational profiles.

In conclusion, this study offers an institution-specific examination of motivational profiles in medical students, highlighting the relevance of entry pathways in shaping self-determined forms of motivation. Graduate-entry students demonstrated consistently higher levels of intrinsic motivation and identified regulation, which may reflect differences in academic background, life experience, or selection processes. These findings suggest the potential value of tailoring educational strategies to the motivational characteristics of distinct student populations. While motivation appeared stable across academic years, further longitudinal and multi-institutional research is needed to assess how curricular structure and broader contextual factors influence motivational trajectories over time. Given the exploratory nature of this study and its cross-sectional design, our conclusions should be interpreted with appropriate caution. Nonetheless, this work contributes to a growing dialogue on how medical schools can better support learner motivation and educational engagement, ultimately informing more inclusive and responsive pedagogical practices.

## Data Availability

The raw data supporting the conclusions of this article will be made available by the authors, without undue reservation.
